# A BDNF/TrKB autocrine loop stimulated by HBZ is involved in HTLV-1-infected cell-survival

**DOI:** 10.1186/1742-4690-11-S1-O57

**Published:** 2014-01-07

**Authors:** Nicholas Polakowski, Marie Terol, Isabelle Nash, Kimson Hoang, Hélène Gazon, Torsten Wurm, Raymond Césaire, Jean-Marie Péloponèse, Jean-Michel Mesnard, Isabelle Lemasson

**Affiliations:** 1 Brody School of Medicine, East Carolina University, Greenville, NC, USA; 2 CPBS, CNRS UMR 5236, Université Montpellier 1, Montpellier, France; 3 Laboratoire de Virologie-Immunologie, Centre Hospitalier et Universitaire de Fort de France, Fort de France, Martinique, France

## 

Brain-Derived Neurotrophic Factor (BDNF) is a neurotrophin that generally promotes neuronal proliferation, survival and plasticity. These effects occur through cellular secretion and subsequent binding of BDNF to its cognate receptor, TrkB, which promotes paracrine and autocrine signaling cascades. A BDNF/TrkB autocrine loop has been implicated in the survival of cells representing several human cancers and is associated with poor prognosis. The HTLV-1 protein, HBZ, is localized to the nucleus where it is capable of deregulating gene expression. In this study, we found that HBZ increases expression of BDNF. There are a variety of transcriptional variants of BDNF, and HBZ increased expression of several of these. Furthermore, multiple transcriptional variants of BDNF were upregulated in HTLV-1-infected cells compared to normal CD4 cells. Expression of the TrkB receptor was also higher in HTLV-1-infected cells than in normal CD4 cells. This trend similarly occurred when comparing ATL patient and healthy donor specimens, with patient cells exhibiting higher levels of BDNF and TrkB expression. TrkB appeared to localize to the cytoplasmic membrane of HTLV-1-infected cells, suggesting a BDNF/TrkB loop contributes to the survival of these cells. This hypothesis was confirmed by chemically blocking the binding of BDNF to TrkB or by inhibiting TrkB signaling, as both treatments led to a significant increase in apoptosis. Together these results suggest that HBZ activates a BDNF/TrkB autocrine loop that enhances survival of HTLV-1-infected cells. This mechanism may ultimately facilitate the survival of ATL cells.

